# Axillary Management for Patients Undergoing Total Mastectomy and a Positive Sentinel Lymph Node: Is Axillary Dissection Necessary?

**DOI:** 10.1002/wjs.12690

**Published:** 2025-07-06

**Authors:** Miranda Addie, Alexander‐Darius Miron, Ericka Iny, Basmah Alhassan, Amina Ferroum, Stephanie M. Wong, Ipshita Prakash, Tarek Hijal, Sarkis Meterissian

**Affiliations:** ^1^ Division of Surgical and Interventional Science Department of Surgery McGill University Montreal Canada; ^2^ Faculty of Pharmacy Université de Montréal Montreal Canada; ^3^ McGill University Medical School Montreal Canada; ^4^ Department of Oncology & Department of Surgery McGill University Health Centre Montreal Canada; ^5^ Segal Cancer Centre Sir Mortimer B. Davis Jewish General Hospital Montreal Canada; ^6^ Department of Oncology Division of Radiation Oncology McGill University Health Centre Montreal Canada

**Keywords:** axillary radiation, completion axillary lymph node dissection, mastectomy, overall survival, positive SLN, sentinel lymph node biopsy

## Abstract

**Purpose:**

We sought to evaluate whether patients with breast cancer who undergo a total mastectomy (TM) can safely forgo a completion axillary lymph node dissection (cALND) in the presence of one to three positive sentinel lymph nodes (SLN+).

**Methods:**

A multicenter retrospective cohort study (2012–2022) was conducted in patients with cT1‐3cN0 who underwent TM with 1–3 SLN+ compared by cALND versus. no further surgery. We compared overall survival (OS) and locoregional recurrence rates (LRR) and investigated whether the omission of cALND altered adjuvant treatment.

**Results:**

In total, the study included 139 patients with SLN+TM, with a mean tumor size of 19.44 mm (SD:10.64); 76% (*n* = 105) of these patients underwent SLNB‐alone. Patients treated by cALND had a younger mean age than those treated by SLNB‐alone (49.5 vs. 56 years and *p* = 0.016). Patients undergoing cALND were more likely to have macrometastatic disease (97% vs. 65% and *p* < 0.001) and extranodal extension (47% vs. 29% and *p* = 0.046). cALND was associated with higher rates of adjuvant chemotherapy (88% vs. 62% and *p* = 0.004). Postmastectomy radiotherapy (PMRT) was similar between groups (79% vs. 82% and *p* = 0.68). At a mean follow‐up of 5.2 years, there was one chest‐wall LRR in the SLNB group, with no axillary recurrences. LRR did not significantly differ with or without cALND (2.9% vs. 1.0% and *p =* 0.4). Five‐year overall survival rates were similar between groups (100% vs. 94% and *p* = 0.2).

**Conclusion:**

We found high OS and low LRR among patients undergoing upfront TM with 1–3 SLN+ without cALND. Completion ALND did not decrease receipt of PMRT but was associated with higher rates of adjuvant chemotherapy. Our findings support the omission of cALND after TM for patients with 1–3 SLN+.

## Graphical abstract

We sought to evaluate whether patents with breast cancer who undergo a total mastectomy (TM) can safely forgo a completion axillary lymph node dissection (cALND) in the presence of one to three positive sentinel lymph nodes (SLN+).

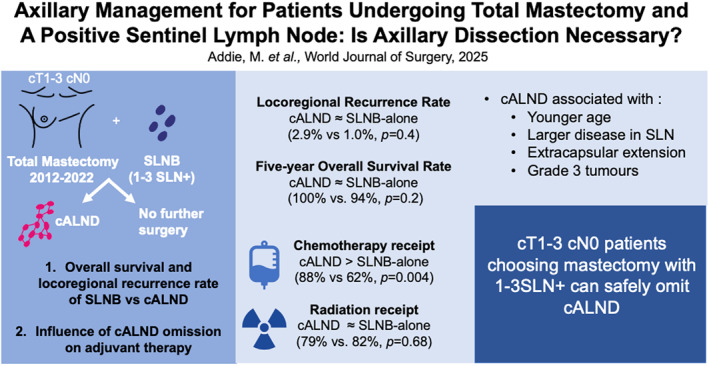

## 1 Introduction

There is ongoing debate regarding optimal axillary management in patients with positive sentinel nodes undergoing total mastectomy (TM). The ACOSOG Z0011 trial, published in 2011, confirmed the safety of omitting cALND in patients with clinical T1–2N0 breast cancer undergoing breast‐conserving surgery (BCS) with one to two positive sentinel lymph nodes (SLN+) [1, 2]. Z0011 did not address similar patients undergoing TM, as they were excluded from the trial. (RNI) [3]. Subsequently, the IBCSG 23‐01 trial, published in 2013, first demonstrated that mastectomy‐treated patients with tumors ≤ 3.5 cm could safely forgo cALND if micrometastatic disease (< 2 mm) were present in 1–2 SLNs [4]. Yet, the controversy persisted, as TM patients comprised only 9% of their cohort [4]. The 2014 publication of the EORTC 10981‐22023 (AMAROS) trial established comparable regional control between cALND and RNI in patients with breast cancer undergoing BCS or mastectomy with 1–3 SLN+ [5, 6]. However, fewer than 20% of the cohort were treated by TM and only 4% had three SLN+, leading to persisting controversy in the axillary management of these patients [5, 6].

To that end, the primary objective of this study was to investigate the influence of cALND on adjuvant therapy in female patients with breast cancer undergoing upfront TM with one to three SLN+. The secondary objective was to compare rates of locoregional recurrence (LRR) and overall survival (OS) between patients treated by SLNB‐alone versus. cALND.

## 2 Methods

### 2.1 Study Population

**FIGURE 1:**
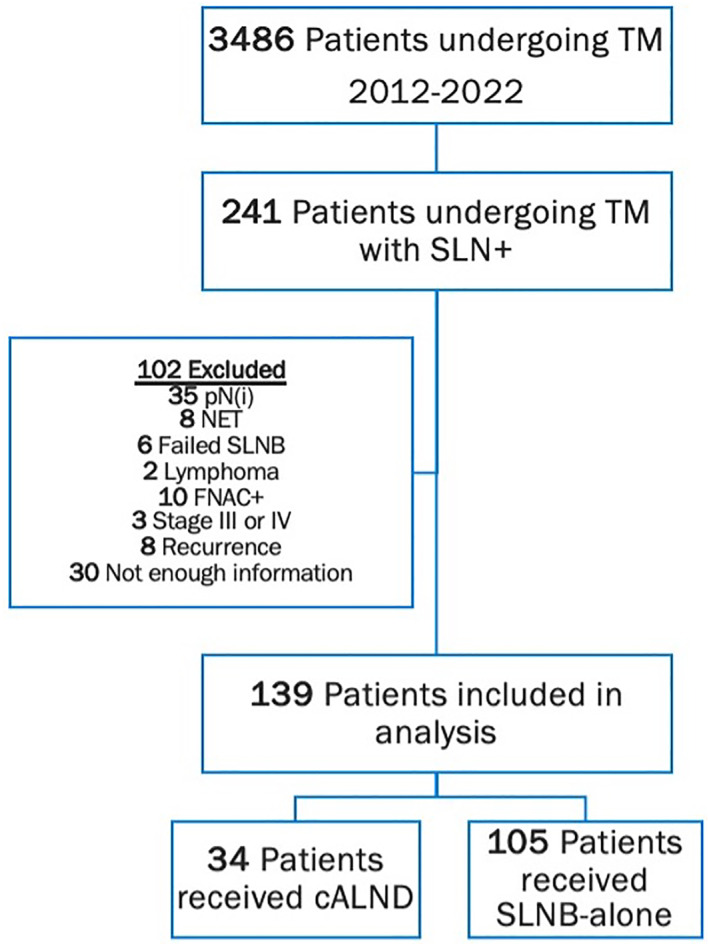
Flowchart of patient inclusion and exclusion criteria.

Following review board authorization, a retrospective chart review was performed at two high‐volume Canadian institutions: the McGill University Health Centre and the Jewish General Hospital. Between 2012 and 2022, a total of 3486 patients underwent total mastectomy (TM) at our institutions. From this cohort, 241 patients with cT1–3 and cN0 disease had 1–3 SLN+. After applying exclusion criteria, 102 patients were removed from the analysis. Exclusions included 35 cases with isolated tumor cells only (pN(i)), eight treated by neoadjuvant endocrine therapy (NET), 6 with failed sentinel lymph node biopsy (SLNB), 2 with lymphoma, 10 with preoperative biopsy‐proven axillary metastasis, 3 with distant metastatic disease at diagnosis, eight presenting with a recurrence, and 30 with insufficient clinical information. The remaining 139 patients comprised the final study population. Abnormal LNs on axillary ultrasound were not an exclusion criterion so long as their fine‐needle aspiration or core biopsy was negative for metastatic carcinoma. The study cohort was divided into two groups: patients treated with cALND or those treated SLNB‐alone with no further axillary surgery. Eligible patients in our study were allowed to participate in the MA39: TailorRT trial, which randomizes patients with biomarker low‐risk node‐positive disease to RNI or no RNI [7]. A detailed breakdown of the study population is presented in Figure 1.

### 2.2 Outcomes and Variable Definitions

The primary outcome of interest was to evaluate whether cALND altered adjuvant treatment compared to SLNB alone. All SLNBs were performed using dual tracers (blue and radioactive dye). Frozen section evaluation of the SLN was routinely omitted after the publications of IBCSG 23‐01 [4] and the AMAROS trial [5] and was replaced with IHC evaluation on permanent sections. Chemotherapy administration and postmastectomy radiotherapy (PMRT) receipt were collected as binary variables. Radiation fields were extracted from radiation oncology treatment plans; the RTOG Breast Cancer Atlas defined target volume boundaries [8]. RNI was denoted as intentional irradiation of the axillary level I–III LNs. Coverage of the axillary level I LNs was defined as no coverage, complete, and incomplete. Complete coverage denoted deliberate inclusion of the axillary vessels at the lateral edge of the pectoralis minor. In contrast, incomplete coverage denoted standard tangential fields covering the lower level I axillary lymph nodes. The secondary outcome of interest was to compare LRR and OS in patients with nodal metastasis undergoing TM with cALND versus SLNB alone. Local recurrences were defined as relapses in the ipsilateral chest wall. *Axillary recurrences* were defined as recurrences in the ipsilateral axillary level I–III nodes. LRR included all ipsilateral local, axillary, and infra‐/supraclavicular nodal recurrences. Distant recurrences were documented when radiological and/or pathological evidence indicated metastatic disease. Five‐year locoregional recurrence‐free survival (LRFS) and distant‐disease‐free survival (DDFS) were defined as the proportion of patients free of locoregional and distant recurrences 5 years from the surgery date. Recurrence and mortality data were censored as of August 1, 2023. Finally, a subgroup analysis of these objectives was performed on patients with macrometastatic disease in the SLN (≥ 2 mm).

### 2.3 Statistical Analysis

Baseline demographic, pathological, and radiologic data were compared between the SLNB‐alone or cALND groups. Patient characteristics were summarized by *n* (%) for categorical variables and mean (standard deviation) or median (interquartile range [IQR]) for continuous variables. The Student's t‐test and the Mann–Whitney *U* test were performed for parametric and nonparametric continuous variables, respectively, Pearson’s chi‐squared or Fisher's exact test was used for categorical variables, including LRR. Five‐year‐LRFS and OS estimates were derived using the Kaplan–Meier survival curves and compared using the log‐rank test. Logistic regression analysis was performed to identify factors associated with the likelihood of chemotherapy in patients with macromestastic disease. Survival estimates were reported along with 95% confidence intervals. A subgroup analysis of patients with macrometastatic disease in the SLN was performed. All analyses were conducted using R Studio software Version 6.1. All statistical tests were two‐sided, with a *p*‐value lower than 0.05 considered statistically significant.

## 3 Results

### 3.1 Clinicopathologic and Radiologic Characteristics

Patient clinicopathological characteristics are summarized in Tables 1 and 2. One hundred thirty‐nine SLN‐positive patients undergoing TM met the eligibility criteria for this study. Of these, 76% (*n* = 105) underwent SLNB alone, whereas 24% (*n* = 34) underwent subsequent cALND. Intraoperative frozen sections (FS) evaluation of SLNs was used in 18% (*n* = 25) of the study cohort. Use of frozen section occurred more frequently in patients who underwent cALND (59%), compared to those who did not (5%) (*p* < 0.001). However, 70% of patients who received FSs were treated between 2012 and 2013, when cALND accounted for 80% of axillary surgeries.

**TABLE 1:** Cohort clinicopathological, treatment, and outcome characteristics (*N* = 139).

Characteristic	Overall (*n* = 139)
Age, yrs.; mean (SD)	56.9 (13.7)
Postmenopausal	83 (61%)
Clinical T stage (cT)	
T1	67 (48%)
T2	40 (29%)
T3	32 (23%)
Clinical T size, mm; mean (SD)	19.4 (10.6)
Multicentric disease, present	39 (28%)
Invasive histologic subtype	
Ductal	88 (63%)
Lobular	35 (25%)
Mixed	16 (12%)
Grade	
Grade 1–2	100 (72%)
Grade 3	39 (28%)
Molecular status	
ER+/pr+/HER2‐	108 (77.7%)
ER+/PR‐/HER2‐	19 (13.7%)
HER2+	11 (7.9%)
TNBC	1 (0.7%)
Pathological T size, mm, median (IQR)	25 (17–37)
Pathological T stage (pT)	
pT1	41 (29%)
pT2	76 (55%)
pT3	22 (16%)
Lymphovascular invasion, present	92 (66%)
#. SLN positive	
1	107 (77%)
2	26 (19%)
3	6 (4%)
# SLN removed, median (IQR)	2.08 (1.1–4.0)
# SLN positive/SLN removed, mean (SD)	0.6 (0.3)
Pathological N stage	
pN1	126 (90%)
pN2	9 (6%)
pN3	4 (3%)
Total # LNs positive; median (IQR)	1.0 (1.0–2.0)
Total # LNs removed; median (IQR)	3 (2.0–7.5)
Extracapsular extension present	46 (33%)
Type of LN deposit	
Macrometastasis	101 (73%)
Micrometastasis	38 (27%)
Largest SLN metastasis (mm); mean (SD)	6.1 (6.0)
Adjuvant therapy received	
Postmastectomy radiotherapy	111 (80%)
Complete axillary radiation	82 (59%)
Chemotherapy	95 (68%)
Endocrine therapy	122 (88%)

**TABLE 2:** Clinicopathological characteristics of patients with or without cALND.

Characteristic	cALND (*n* = 34)	SLNB‐alone (*n* = 105)	*p*‐value
Age, yrs.; mean (SD)	51.71 (12.0)	58.5 (13.9)	**0.007**
Postmenopausal	17 (52%)	66 (63%)	0.22
Clinical T stage (cT)			0.82
T1	15 (44%)	52 (49.5%)	
T2	10 (29%)	30 (28.5%)	
T3	9 (26%)	23 (22%)	
Clinical T size, mm; mean (SD)	20.3 (11.7)	19.2 (10.3)	0.69
Multicentric disease, present	11 (32%)	28 (27%)	0.52
Invasive histologic subtype			0.51
Ductal	20 (59%)	68 (65%)	
Lobular	11 (32%)	24 (23%)	
Mixed	3 (9%)	13 (12%)	
Grade			0.81
Grade 1–2	25 (74%)	75 (71%)	
Grade 3	9 (26%)	30 (29%)	
Molecular status			0.21
ER+/pr+/HER2‐	25 (74%)	83 (79%)	
ER+/PR‐/HER2‐	4 (12%)	15 (14%)	
HER2+	4 (12%)	7 (7%)	
TNBC	1 (2%)	0 (0%)	
Pathological T size, mm, median (IQR)	30 (22–44)	25 (16–35)	**0.042**
Pathological T stage (pT)			0.14
pT1	6 (18%)	35 (33.3%)	
pT2	20 (59%)	56 (53.3%)	
pT3	8 (23%)	14 (13.3%)	
Lymphovascular invasion, present	27 (79%)	65 (62%)	0.061
No. SLN positive			0.86
1	26 (76%)	81 (77%)	
2	6 (18%)	20 (19%)	
3	2 (6%)	4 (4%)	
#. SLN removed, median (IQC)	1.6 (1.1–3.0)	2.1 (2.0–4.0)	**0.027**
SLN positive/SLN removed, mean (SD)	0.8 (0.3)	0.6 (0.3)	**0.007**
Pathological N stage			**<** **0.001**
pN1	21 (62%)	105 (100%)	
pN2	9 (26%)	0 (0%)	
pN3	4 (12%)	0 (0%)	
Total no. LNs positive; mean (SD)	5.0 (7.6)	1.3 (0.6)	**0.008**
Total no. LNs removed; median (IQR)	13 (10.3–16.8)	3 (2.0–4.0)	**<** **0.001**
Extracapsular extension, present	16 (47%)	30 (29%)	**0.046**
Type of LN deposit			**<** **0.001**
Macrometastasis	33 (97%)	68 (65%)	
Micrometastasis	1 (3%)	37 (35%)	
Largest SLN metastasis (mm); mean (SD)	7.8 (4.7)	5.52 (6.3)	**0.045**
Adjuvant therapy received			
Postmastectomy radiotherapy	28 (82%)	83 (79%)	0.68
Axillary radiation	15 (44%)	67 (64%)	**0.042**
Chemotherapy	30 (88%)	65 (62%)	**0.004**
Endocrine therapy	30 (88%)	92 (88%)	1

*Note:* *p*‐values that are bolded signify statistical significance (*p* < 0.05).

Patients were statistically younger in the cALND group, with a mean age of 51.7 years (SD:12.0 yrs), compared to 58.6 years (SD:13.9 yrs) in the SLNB alone group (*p* = 0.016). The presence of one or more abnormal axillary LNs did not significantly differ between groups, occurring in 32% in the cALND group and 19% in the SLNB alone group (*p* = 0.11). However, patients undergoing cALND were more likely to have had a preoperative axillary lymph node FNA or core biopsy (32% vs. 10%, and *p* = 0.045). Preoperatively, the groups did not differ significantly in other clinicopathologic variables. A trend suggested that patients receiving cALND were primarily premenopausal, but this was not statistically significant when including patients with micrometastatic SLN.

Most patients had pT2 disease. The median tumor size on final pathology was slightly larger at 30 mm for patients treated with cALND compared to 25 mm for those who were not (*p* = 0.042). More patients who underwent cALND (79%) had lymphovascular invasion compared to those who underwent SLNB (62%), although not statistically significant (*p* = 0.061). The number of SLN+ did not differ between groups, but patients undergoing cALND were more likely to have macrometastatic disease (97% vs. 65% and < 0.001), extracapsular extension (47% vs. 29% and *p* = 0.046), and fewer SLN removed (1.55 vs. 2.09 and *p* = 0.027). Specifically, only one patient (3%) in the cALND had purely micrometastatic disease compared to 35% of those undergoing SLNB alone. The mean SLN ratio, defined as the number of positive SLN of the total number of SLN, was higher in the patients treated by cALND (0.8 vs. 0.6 and *p* < 0.001). There were no differences in the patient characteristics among those with 1, 2, and 3 SLN+.

### 3.2 Influence of cALND on Adjuvant Treatment

Most of the study population (68%) underwent adjuvant chemotherapy (Table 1). However, cALND was associated with higher rates of adjuvant chemotherapy (88% vs. 62%, *p* = 0.004, and Table 2). Most (88%) received anthracycline‐based chemotherapy, 4% received platinum‐based chemotherapy and 6% received taxane‐only chemotherapy. There were no differences in the chemotherapy regimen by axillary surgery. The use of endocrine therapy was high and the same for both groups.

The use of PMRT was similar for those with and without cALND (82% vs. Seventy‐nine percent and *p* = 0.68). Overall, patients who received radiation therapy were likely to have larger tumor size at presentation (16.2 mm SD: 9.0 vs. 20.3 mm SD: 10.9 and *p* = 0.048) and larger size of metastatic deposit in the LNs at surgery (6.6 mm SD: 6.3 vs. 3.9 mm SD: 3.8 and *p* = 0.004). cALND was associated with a lower rate of axillary radiation compared to SLNB alone (44% vs. 64% and *p* = 0.042). Twenty‐eight patients in our study did not undergo PMRT, of which 25% (*n* = 7) declined, 14% (*n* = 4) had distant metastatic disease after surgery, and 61% (*n* = 17) were believed to receive minimal benefit from PMRT (small tumor size, single positive SLN, and no extracapsular extension). The radiation field coverage of the level I axillary lymph nodes differed significantly between the groups (*p* < 0.001). Complete coverage of the axilla occurred in 15 (63%) patients in the cALND group compared to 71 (92%) in the SLNB‐alone group. Radiation dose consisted of 50–50.4 Gy in 25–28 fractions in 58% of the cohort, hypofractionation (40.05–42.4 Gy in 15–16 fractions) in 36%, and ultra‐hypofractionation (27.5–28.5 Gy in 5 fractions) in 6%. The radiation field characteristics can be found in Table 3.

**TABLE 3:** Radiation field characteristics among patients treated by PMRT with or without cALND (*n* = 111).

Radiation fields	cALND (*n* = 28)	SLNB‐alone (*n* = 83)	*p*‐value
Location			**0.019**
Chest wall	1 (4%)	6 (7.5%)	
Chest wall + IMN	0 (0%)	2 (2.5%)	
Chest wall + SC	8 (33%)	5 (6%)	
Chest wall + SC + axilla	7 (2%)	36 (45%)	
Chest wall + SC + axilla + IMN	8 (33%)	31 (39%)	
Missing	4	3	
Axillary level I fields			**<** **0.001**
No coverage	0 (0%)	1 (1%)	
Complete	15 (62%)	71 (92%)	
Partial	9 (38%)	5 (7%)	
Missing	4	3	
Chest wall boost			0.21
Yes	10 (38%)	19 (25%)	
No	16 (62%)	58 (75%)	
Missing	2	6	
Dose/Fractions			**<** **0.001**
50–50.4 Gy/25–28 fractions	24 (92%)	38 (47%)	
40.05–42.4 Gy/15–16 fractions	2 (8%)	37 (46%)	
27.5–28.5 Gy/5 fractions	0 (0%)	6 (7%)	
Missing	2	2	

*Note:* *p*‐values that are bolded signify statistical significance (*p* < 0.05)

### 3.3 Influence of cALND on Survival Outcomes

**FIGURE 2:**
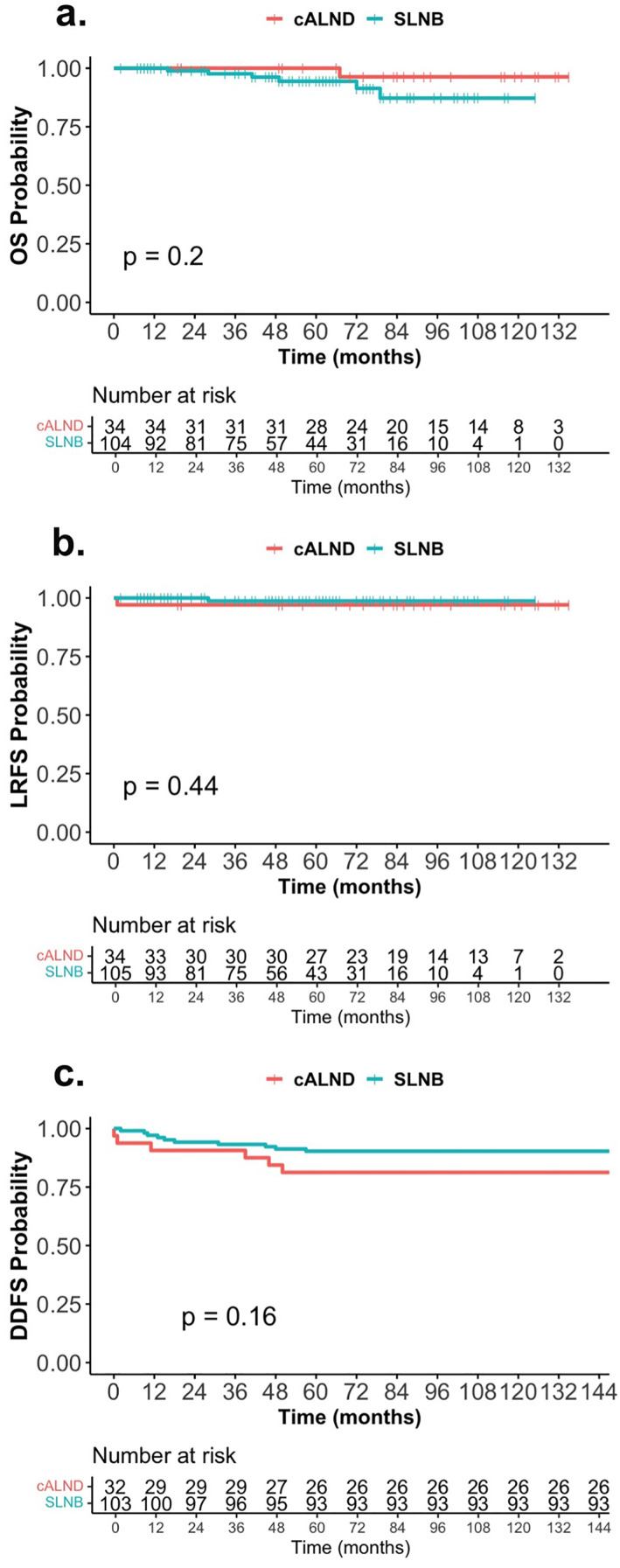
Kaplan–Meier survival curves by axillary surgery: (a) overall survival (OS), (b) locoregional recurrence‐free survival (LRFS), and (c) distant disease‐free survival (DDFS).

The mean follow‐up time for the entire study cohort was 61.8 months (SD: 35.5 years). However, patients in the cALND group had a mean follow‐up duration of 90.8 months compared to 52.5 months in the SLNB‐alone group (*p* < 0.001). The 5‐year OS was high and similar for both groups: 100% in the cALND group (95% CI: 100%–100%) and 94% in the SLNB‐alone group (95% CI: 89%–100%) (*p* = 0.2) (Figure 2a). The 5‐year LRFS was 97.1% (95% CI: 91.5–100) versus. 98.7% (95% CI: 96.3–100) for patients with and without cALND, respectively (*p* = 0.44) (Figure 2b). LRR did not significantly differ in patients with and without cALND (2.9% vs. 1.0%, *p* = 0.43). The 5‐year DDFS was 82% (95% CI: 70–96) in the cALND group and 87% (95% CI: 80–95) in the SLNB‐alone group (*p* = 0.16) (Figure 2c). Five‐year survival estimates derived from the Kaplan–Meier analysis and log‐rank tests can be found in Table 4.

**TABLE 4:** 5‐year survival estimates with or without cALND.

Outcome	cALND (*n* = 34)	SLNB‐alone (*n* = 105)	*p*‐value
5‐year OS (95% CI)	100% (100%, 100%)	94% (89%, 100%)	0.2
5‐year LRFS (95% CI)	97% (92%, 100%)	99% (96%, 100%)	0.44
5‐year DDFS (95% CI)	82% (70%, 96%)	87% (80%, 95%)	0.44

### 3.4 Subgroup Analysis in Patients With Macrometastatic Disease

Macromestastic disease in the SLN occurred in 101 patients: 33 underwent cALND and 68 underwent SLNB alone. Patients who underwent cALND were more likely to be premenopausal (47% vs. 26% and *p* = 0.043) and younger (49 years (IQR: 44–60) versus. 61.5 years (IQR: 29–73) and *p* = 0.002). Multivariable analysis revealed that patients treated between 2014 and 2022 had significantly lower odds of undergoing cALND compared to those treated between 2012 and 2013 (OR = 0.01, 95% CI: 0.00–0.05, and *p* < 0.001). Patients over 50 years were associated with lower odds of cALND (OR = 0.94, 95% CI: 0.90–0.99, and *p* = 0.019), whereas factors, such as number of SLN+, tumor grade and histology, lymphovascular invasion and extracapsular extension, did not reach statistical significance.

Radiation receipt did not differ (85% in both groups). Again, patients with cALND were more likely to receive adjuvant chemotherapy (88% vs. 65% and *p* = 0.015). In assessing the predictors of adjuvant chemotherapy among these patients, younger individuals (under 50 years) exhibited significantly greater odds of receiving chemotherapy (OR = 6.19, 95% CI: 1.78–29.4, and *p* = 0.009). Conversely, those with SLNB‐alone showed decreased odds of receiving chemotherapy (OR = 0.23, 95% CI: 0.06–0.77, and *p* = 0.026). A higher tumor grade (Grade 3) correlated with increased odds of chemotherapy (OR = 5.69, 95% CI: 1.74–23.2, and *p* = 0.007). Factors, such as extranodal extension (OR = 0.68, 95% CI: 0.23–1.96, and *p* = 0.47) and lymphovascular invasion (OR = 0.82, 95% CI: 0.26–2.44, and *p* = 0.73) did not show a significant association with chemotherapy administration. No differences were seen in OS (100% vs. 94% and *p* = 0.22), DDFS (87% vs. 91% and *p* = 0.89), or LRFS (100% vs. 100% and *p* = 0.25).

## 4 Discussion

The decision to perform cALND in patients undergoing total mastectomy and having 1–3 positive sentinel nodes remains controversial. This study included 139 SLN‐positive patients treated by total mastectomy (TM) comparing those who underwent cALND (24%) versus SLNB‐alone (76%). There were no significant differences in axillary management practices between surgeons within this study. The considerable decline in cALND rates after 2013 highlights the paradigm shift in axillary management, influenced by landmark trials such as Z0011 [1, 2], IBCSG 23‐01 [4], and AMAROS [5, 6] trials. Most cALND procedures (65%) were performed at the index surgery.

The first objective was to evaluate whether performing a cALND altered the use of postoperative adjuvant therapy. PMRT and axillary radiation have been reported to provide noninferior survival compared to cALND [9, 10, 11, 12]. In 2016, guidelines recommended PMRT for early breast cancer (T1–2) with 1–3 positive lymph nodes due to enhanced local control and reduced mortality [13]. Our results demonstrate that most patients received adjuvant radiation regardless of axillary surgery. RNI fields covering axillary levels I–III lymph nodes were more common in patients with SLNB‐alone than those who underwent cALND. The high frequency of PMRT in our study contrasted with a recent South Korean study that found 30.4% of patients with cALND and 26% of SLNB‐alone received PMRT [14].

We found that patients undergoing cALND were more likely to undergo chemotherapy compared to patients who underwent SLNB alone. These findings align with similar retrospective studies, which reported higher rates of adjuvant chemotherapy in patients who received cALND than those who did not [12, 15]. Chemotherapy receipt in our SLNB‐only group was similar to the Z0011 (61%) [1], the SENOMAC (65%) [16], and the SINODAR ONE subanalysis (52%) [17].

The second objective of this study was to evaluate the OS and LRR associated with SLNB‐alone versus cALND. We demonstrated comparable OS and extremely low LRR associated with SLNB‐alone compared to cALND. Both groups had only one LRR, but the SLNB‐only group had a chest wall recurrence and no axillary recurrences. These findings support results from prior studies indicating low recurrence rates in patients with SLN+ mastectomy, regardless of axillary surgery [4, 5, 15, 16, 18]. However, an important consideration when considering SLNB‐alone is the size of the metastatic disease in the SLN. The IBCSG 23‐01 trial (performed between 2001 and 2010) randomized patients with 1–2 micrometastatic SLN to cALND or no cALND [4]. The trial involved 86 patients with mastectomy, revealing that omitting cALND was noninferior, with a hazard ratio of 0.52 (0.09–3.10). At 10 years, axillary recurrence rates were low: cALND 0.2% versus. 1.2% [19]. It is unclear how many patients received adjuvant radiation but 92% underwent adjuvant chemotherapy [19]. Our study supports the safety of omitting cALND for patients with micrometastatic disease in the SLN, as there were no locoregional recurrences in these patients.

Omitting cALND for patients with macrometastatic disease remains controversial, representing 73% of our cohort. The SINODAR‐ONE trial (2015–2020) demonstrated equivalent survival outcomes regardless of cALND in patients with 1–2 positive SLNs, though only 22% underwent TM [20]. Their subsequent subanalysis (*n* = 218) of mastectomy patients confirmed excellent survival rates (97.8% vs. 98.7%) [17]. The SENOMAC trial (2015–2021) expanded these findings to include patients with extracapsular extension and cT3 disease [16]. Although both trials followed European radiation protocols without standardization, their results collectively supported our institutional shift from routine cALND after the publication of AMAROS in 2014 [5].

Macrometastatic disease was found in the SLN of 101 patients in this study. cALND was more frequent in younger premenopausal women who had surgery between 2012 and 2014 and went on to receive chemotherapy. In these patients, chemotherapy decisions were independently predicted by age < 50 years (OR = 6.19) and high‐grade tumors (OR = 5.69) but not by extranodal extension or lymphovascular invasion. Higher chemotherapy rates in patients treated by cALND may also be attributed to the fact that these patients upgraded to pN2–3. The absence of survival differences between patients treated with or without cALND supports the safety of SLNB‐alone in patients with 1–3 SLN+ treated by mastectomy, consistent with prior investigations [4, 15, 16, 17, 21, 22, 23]. After excluding patients treated between 2012 and 2013, the survival outcomes and adjuvant therapy did not change, supporting the strength of these findings. Further evidence on the safety of adjuvant therapy alone compared to cALND or RNI will be provided from the ongoing POSNOC trial [24].

The limitations of our study arise from its retrospective design, affecting result interpretation. Treatments were conducted at two institutions over a decade, resulting in varied hypofractionation regimens. Despite this, survival outcomes were consistent, with local recurrence rates reported at different sites. Most patients did not undergo cALND, reflecting real‐world trends following major axillary management trials. Although the group distribution is uneven and the study population is small, this study contrasts with earlier studies that included cohorts where few patients omitted cALND [9, 15, 22, 25, 26]. Snow et al. and FitzSullivan et al. reported that among those SLN+ mastectomy‐treated patients, only 17% (*n* = 32) and 13% (*n* = 70) of these patients, respectively, did not receive cALND [15, 22]. Since 91% of patients had HR+/HER2‐disease, which typically has longer recurrence intervals, the lack of axillary recurrences in the SLNB‐only group warrants further investigation. All patients with HER2+ and TNBC had tumors smaller than 1 cm, as patients with larger tumors were triaged to neoadjuvant chemotherapy and no recurrences occurred in these patients.

## 5 Conclusion

We found high OS and low LRR among female patients undergoing upfront mastectomy with 1–3 SLN+ without cALND. Completion ALND did not decrease receipt of postmastectomy radiation, but increased targeted axillary radiation and higher rates of adjuvant chemotherapy. Our findings suggest that patients choosing mastectomy with low or intermediate grade disease with low nodal burden (1–2SLN+) can safely omit cALND if they received targeted axillary radiation and PMRT.

## Author Contributions

**M.A.:** conceptualization, methodology, data acquisition, validation, statistical analysis, writing – original draft, review and editing, manuscript review. **A.‐D.M., E.I., A.F.:** data acquisition, manuscript review. **B.A.:** conceptualization, manuscript review. **I.P., S.M.W.:** data analysis and interpretation, writing – review and editing, manuscript review. **T.H.:** conceptualization, methodology, validation, supervision, data analysis, interpretation, writing – review and editing. **S.M.:** conceptualization, supervision, methodology, validation, data analysis, interpretation, writing – original draft, review and editing, manuscript review.

## Ethics Statement

The Institutional Review Board approved this study, and Director of Professional Services approval consent was obtained in lieu of individual patient consent.

## Conflicts of Interest

The authors declare no conflicts of interest.

## Data Availability

The data that support the findings of this study are available from the corresponding author upon request. The data are not publicly available due to privacy or ethical restrictions.
